# Syntactic Complexity Phenomena Are Better Explained Without Empty Elements Mediating Long‐Distance Dependencies

**DOI:** 10.1111/cogs.70088

**Published:** 2025-08-17

**Authors:** Yanis da Cunha, Edward Gibson

**Affiliations:** ^1^ Department of Romance Studies University of Graz; ^2^ Department of Brain and Cognitive Sciences Massachusetts Institute of Technology

**Keywords:** Sentence processing, Syntax, Acceptability judgment, Empty categories, Long distance dependencies, Filler‐gap constructions

## Abstract

We report the results of two acceptability judgment experiments on English materials, which were designed in order to help disentangle predictions of syntactic theories with transformations from nontransformational theories. The materials in these experiments were motivated from examples from Pickering & Barry (1991), who provided intuitive evidence that there is little processing cost for connecting a fronted prepositional phrase to its verb, even if it is the second postverbal argument of a verb in the declarative form. For example, the PP *on which* connects to the verb *put* in the sentence *This is the saucer on which Mary put the cup into which she poured the milk*. If there is a transformation of phrases from declarative structures to interrogative structures (as proposed in Chomsky (1957) and all versions of related theories since), then there is a long‐distance connection between the fronted PP and its base position following the NP object, for example, *the cup into which she poured the milk*, which is not complete until the end of the sentence. In contrast, in a theory without transformations, the PP can be directly associated with its role‐assigning verb *put* when this verb is encountered. If there is cost for processing making dependency connections that is proportional to their distances, then transformational theories predict a large processing cost for this kind of structure, relative to controls. In contrast, nontransformational theories predict no large cost. The results of the two rating experiments consistently supported the predictions of the non‐transformational theories relative to those of the transformational theories. We argue that, in line with other current evidence, the nontransformational theories appear to better support the available empirical data.

## 1 Introduction

Going back to Chomsky (1957, 1965), many syntax researchers have proposed that there is a base structure—called **deep structure** by Chomsky (1965)—and transformations or “movement” operations that apply to the base structure, in order to result in a surface form. For example, the auxiliary verb *might* in the interrogative yes‐no question in (1a) is proposed to move from its declarative base position, as in (1b):^1^

(1)
  a. [Might]_*i*_ Ollie _*i*_ chase the squirrel?
  b. Ollie might chase the squirrel.

Another proposed syntactic transformation involves moving the noun phrase (NP) object of an active verb to the subject position of the passive form of the same verb (Chomsky, 1957, 1965). For example, the object NP *the squirrel* is proposed to start in object position in (2a) and then move to the subject of the passive in (2b):

(2)
  a. Ollie chased [the squirrel].
  b. [The squirrel]_*i*_ was chased _*i*_.

A third proposed syntactic transformation involves moving an object of a verb in a declarative clause to the front of that clause in a fronting construction, such as a wh‐question, relative clause, or cleft. For example, the object NP *what* starts in the object position of the verb *chase* in (3)—the same position as the squirrel in (2a)—and moves to a position at the front of the clause:

(3) [What]_*i*_ did Ollie chase_*i*_ ?

A potential advantage of the transformation‐based proposal is that three very different phenomena—subject inversion, passivization, wh‐fronting —appear to be derived by the same kind of operation: a transformational movement.

### 1.1 The difficulty of learning transformations: Structure dependence

Chomsky (1971) later noted that a transformational theory is difficult—perhaps impossible—for a child to learn, based only on the input that is available. In particular, Chomsky (1971) observed that it is difficult to learn which position should be fronted in a complex sentence like (4), in order to form a yes‐no‐question:

(4) The dog that is in the corner is hungry.

If we front the first of these, we end up with a non‐English question:

(5) * [Is]_*i*_ the dog that _*i*_ in the corner is hungry?

We need to front the second of these from (4)—the one associated with the main clause—in order to make a grammatical question from this source:

(6) [Is]_*i*_ the dog that is in the corner _*i*_ hungry?

Chomsky referred to this property as **structure dependence**: English speakers front the auxiliary associated with the main clause of the sentence. He noted that this movement process would be difficult or perhaps impossible for a child to learn because they would need to be exposed to an example like (6) in order to learn the correct movement operation. But complex materials like (6) are exceedingly rare: so rare that the child may never encounter one. Furthermore, Chomsky hypothesized that people would never make errors as in (5). Hence, Chomsky argued that structure dependence of the transformation from declarative position to the interrogative position must be innate.

We will argue below that this argument is flawed because it presumes that transformations must be part of the grammar: the movement of the auxiliary from declarative position to interrogative position. If there were no such transformations, then the grammar may be learnable (cf. Perfors, Tenenbaum, & Regier, 2006; Sag et al., 2020; see also Peters and Ritchie (1973) for a discussion of how transformational grammars can generate any recursively enumerable set, which has the consequence that they are difficult to learn).

Indeed, Chomsky is correct that learning a set of syntactic rules which includes the possibility that elements can move around is more difficult than if movement is not possible.^2^ Summarizing from Gibson (2025), let us consider (1b) and two possible representations that one might need to learn, depending on the syntactic theory: a nontransformational dependency grammar analysis (7), or a transformation‐based phrase structure analysis (8), using Chomsky's “Barriers” framework (Chomsky, 1986), the precursor to the “Minimalist Program” (Chomsky, 1993). The external argument NP *Ollie* starts inside the VP, and moves to the specifier subject position of IP (Fukui & Speas, 1986; Koopman & Sportiche, 1991) and the auxiliary *might* starts as a head I and moves to the head position C:

 
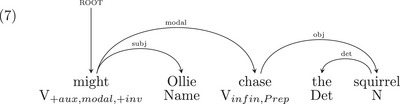


 
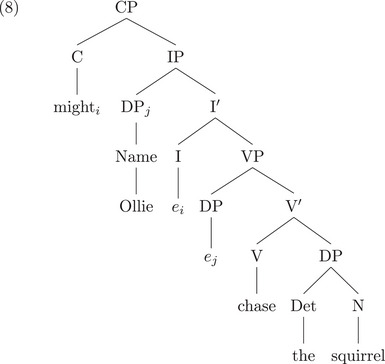


We can simplify the phrase structure in (8) to the dependency representation in (9) below, which contains all the headedness information in the phrase structure:

 
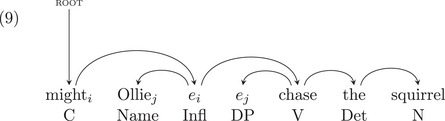


All the information in (7) is included in (9), but with more information in (9). So, learning structures like those in (7) is strictly easier than learning structures as in (9).

Let us first assume that the learner can learn the words and morphemes, such that the learning problem amounts to learning how these words connect to one another in the dependency rules: the tree structure. If there are only two words *a* and *b*, then there are only two possibilities: *a* is a dependent of *b*, or *b* is *a* dependent of *a*. For a three word sentence, the possibilities multiply quickly: any of the three words might be the head of the whole sentence, as shown in (10).

 
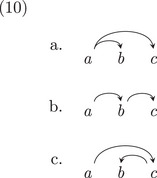


Hence, there are nine possible dependency structures for a set of three words. In general, there are $n^{n-1}$ possible structures for a sequence of *n* words (Caminiti, Finocchi, & Petreschi, 2007; Prüfer, 1918). This is large number of possible structures, but perhaps, we can learn the structures for smaller parts of sentences first before figuring out the structure of a longer sentences, for example, Elman (1993). So, for a four‐word sequence like *Ollie will chase Lana*, we have 64 possible structures. And for a five‐word sequence like *Will Ollie chase the squirrel*, we have 625 possible structures.

Let us now consider the learning problem for the transformation‐based grammar. Not only do we need to learn the headedness structure of a directed acyclic graph, but we also have to allow for the possibility that any of the words started out somewhere else, and moved some number of times. Let us consider the simpler possibility that each word moved at most once. (Our simplifying assumptions will, therefore, *underestimate* the search involved for transformed structures in two ways: (1) it is not only words that can move, but whole constituents; and (2) they may move more than once in each structure.) For an *n*‐word sequence, we increase the search space from $n^{n-1}$ possible dependency structures by another factor of $n^{n}$: Each word can either stay where it is, or move to each of *n* positions. (If the moved element $a_{0}$ is immediately beside its root element a, this is “vacuous” movement: movement which is not across any words. We consider this equivalent to the unmoved variant.) The number of structures $S_{NoTransformations}$ to be considered for a base dependency analysis is in (11a); the number of structures $S_{Transformations}$ to be considered for an analysis with transformations of only one per word is in (11b):

(11)
  a. $S_{NoTransformations}=n^{n-1}$
  b. $S_{Transformations}=n^{n-1}\times n^{n}=n^{2n-1}$

For example, in each of the nine structures for three‐word sequences, each word can move to three other positions: this is 27 possibilities for each of the source structures. We provide three of these below, for the first structure in (10), where *b* and *c* are unmoved.

 
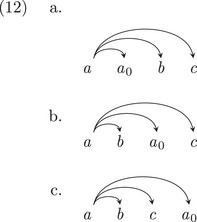


Thus, for a three‐word sequence, we increase the search space from nine possible dependency structures by a factor of 27, to 243 possible structures. And, for a four‐word sequence, we increase the search space from 64 possible dependency structures by a factor of 256 to 16,384 possible structures. For a sequence of five words, the search goes from 625 possible structures to 1,953,125 structures. This is an astronomical search. So, Chomsky is indeed right that his particular grammar is difficult to learn. In contrast, the dependency grammar structures—with no movement or empty elements— are far easier to learn (Gibson, 2025).

### 1.2 Nontransformational approaches to displacement phenomena

An alternative that Chomsky did not consider is that the grammar might not contain transformations. This would greatly simplify the learning problem. How then can we implement the idea that an auxiliary verb often has two possible positions, one for its declarative meaning, and one for its interrogative meaning? One way of implementing such an idea is through lexical featural ambiguity and feature passing (Bresnan, 1982a; Sag et al., 2020; Steedman & Baldridge, 2011). For example, consider the case of auxiliary verb transformations, such as involving a modal verb like *will* in (1b). The word *will* could then have two separate related lexical entries: one which expects a subject noun before it and an infinitive verb to follow when it is declarative (13a), and another which expects a subject noun following it and an infinitive verb to follow, when it is interrogative (13b) (Abeillé & Rambow, 2000; Sag & Fodor, 1994; Steedman, 2001):

 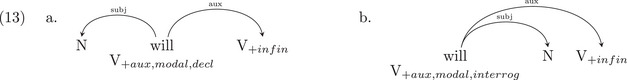


This can be implemented as two related lexical entries (as above), or as one lexical entry that has an underspecified feature matching either the declarative or interrogative usages.

It is possible that Chomsky may have thought that it was inefficient to have two lexical entries for an auxiliary verb (or an active vs. passive verb. or for declarative vs. interrogative argument structures). While it is indeed slightly more complex to have an extra lexical entry, we first should note that common words typically have many lexical entries, corresponding to many different senses. A high‐frequency English word like *take* or *run* has more than 30 different senses in any standard dictionary. So, an additional syntactic possibility does not seem especially inefficient, for any of these common English words. But, more importantly, as observed above, the grammar is astronomically more complex when transformations are permitted. So, the efficiency argument goes in the direction favoring the nontransformational accounts over transformational ones.

### 1.3 Empirical consequences of nontransformational approaches

In addition to comparing the complexity of transformational and non‐transformational grammars, we can also compare the empirical consequences of each kind of framework. Sag et al. (2020) provide several kinds of examples that support the nontransformational account over the transformational one with respect to the English auxiliary system. Most generally, the transformational approach predicts that all auxiliary verbs should be possible in both the declarative and the interrogative versions, with the same meaning, because the interrogative is derived from the declarative. In contrast, the standard nontransformational approach is a usage‐based approach, whereby the learner needs to be exposed to the usage of each of the declarative and interrogative usages before they acquire them. There might still be a rule— whereby the interrogative form is formed from the declarative—but this is only if the learner is exposed to the interrogative version. Hence, this account is compatible with there being examples that are only used in one version or the other.

Acceptability data from English speakers support the predictions of the nontransformational account over the transformational account. In particular, there are English auxiliary forms that are only possible in the declarative (14–17), and forms that are only possible in the interrogative (18):

(14)
  a. I ought to go to the party.
  b. * Ought I to go to the party?

(15)
  a. I better to go to the party.
  b. * Better I to go to the party?

(16)
  a. Ev must be in her office.
  b. * Must Ev be in her office? (when acceptable, different meaning of *must* in declarative)

(17)
  a. Ev may be in her office.
  b. * May Ev be in her office? (when acceptable, different meaning of *may* in declarative)

(18)
  a. Aren't I invited to the party?
  b. * I aren't invited to the party.

Consider the low‐frequency auxiliary verb *ought* in (14). This auxiliary is only possible for most American and Canadian English speakers in the declarative.^3^ The interrogative is simply not possible. The same is true for several other English auxiliary verbs. The auxiliary verb *may* is possible for American and Canadian speakers in the interrogative (17), but only for an asking‐permission interpretation, not for a possibility interpretation, whereas the declarative prefers a possibility interpretation. These usage data support the nontransformational account over the transformational account. Such data show that one can actually *benefit* from the lexical analysis to avoid overgeneration. Making the lexicon more complex when simplifying the grammar thus leads to a better account of lexically conditioned phenomena. Chomsky (1970) also argued along these lines regarding the nominalization rule (e.g., *destroy* → *destruction*), suggesting that one should remove it from the transformational component because of its numerous complexities and its lexical dependence (see also Dowty (1978) on lexically governed transformations for a similar argumentation).

Bresnan (1982a, 1982b) proposes a lexical rule approach to the passive for similar reasons. In particular, there are many English verbs that are only possible in the active form:

(19)
  a. Ollie had a squirrel.
  b. * A squirrel was had by Ollie.

(20)
  a. The house lacked a garage.
  b. * A garage was lacked by the house.

(21)
  a. The dress suited Lana.
  b. * Lana was suited by the dress.

And conversely, some passives have no active counterpart (22) as shown by Bresnan, Asudeh, Toivonen, & Wechsler (2015a, section 2). Cases like those are called “movement paradoxes” by the authors: how can one derive a passive sentence if its putative active source is ungrammatical?

(22)
  a. * This theory captures that languages are learnable. (example taken from Bresnan et al., 2015a, p. 13)
  b. That languages are learnable is captured by the theory.

If there were a transformational rule, it would apply to all verbs. A lexical construction‐based approach explains the observed usage data more easily.

### 1.4 Filler‐gap constructions

In the current paper, we investigate some ramifications of transformational versus nontransformational accounts with respect to filler‐gap constructions in English. Filler‐gap constructions represent a class of constructions, where a phrase located in a noncanonical position, called the *filler*, comes with an nonlexicalized canonical position, called the *gap* ^4^ (Fodor, 1978). Sentence (23) illustrates these notions with a *wh*‐interrogative clause: the filler is *which book*, while the gap is the unoccupied position after *read*.

(23) [Which book]_*filler*_ did Mary say you have to read – _*gap*_ tomorrow?

Going back to early work by Chomsky (1957, 1965), transformational frameworks have assumed that gaps are not mere empty positions, but that they are actually occupied by syntactic elements, such as “traces” in the Government & Binding theory (GB) (Chomsky, 1981), or “copies” in the Minimalist Program (Chomsky, 1995). For example, sentence (23) can be analyzed as in (24), where the gap is represented as an unpronounced copy of the filler. To generate such a copy, the Minimalist analysis assumes an operation called *Move* (or *internal Merge*), which moves *wh*‐phrases (and other kinds of elements) from their canonical position to the noncanonical position, leaving a copy behind. Because of this copy, the verb *read* can maintain a local argumental relation with *which book*, while the phrase is pronounced earlier in the sentence.

(24) [Which book] did Mary say you have to read <which book> tomorrow?

By contrast, nontransformational frameworks have adopted movement‐free analyses of filler‐gap constructions. For example, Head‐driven Phrase Structure Grammar (HPSG, Sag, 2010; Borsley & Crysmann, 2021) links the filler to its head by the percolation of a nonlocal feature (called slash, Gazdar, Klein, Pullum, & Sag, 1985). In Lexical Functional Grammar (LFG, Bresnan, Asudeh, Toivonen, & Wechsler, 2015b; Dalrymple, Lowe, & Mycock, 2019), the filler is licensed through functional uncertainty (Kaplan & Zaenen, 1989), which assigns the filler the syntactic function that its head misses. In Combinatory Categorial Grammar (CCG, Steedman, 2001; Steedman and Baldridge, 2011), fillers are assigned specific categories that make them select clauses with missing phrases. In all cases, the analyses yield structures without empty categories. Proponents of such frameworks have argued that empty categories are theory‐internal artifacts leading to more complex and sometimes inadequate structures (Dalrymple, Kaplan, Maxwell, & Zaenen, 1995 cf. Bresnan, 1978; Bresnan & Kaplan, 1984; Steedman, 2001; see the discussion of the *“wanna”* contraction in Sag and Fodor (1994) or weak crossover in Bresnan (1995, 1998); Dalrymple, Kaplan, King, & Butt (2001)). In any case, linguists and psycholinguists have also used language processing data to help decide between theories with and without empty elements associated with movement.

### 1.5 Filler‐gap constructions in psycholinguistics

Filler‐gap constructions involve dependencies between elements at an arbitrary distance apart. Unlike local dependencies (25a), nonlocal dependencies (25b) require the use of memory to maintain a filler representation and a predictive processing about how this filler has to be interpreted. This filler‐driven processing is sometimes called Active Dependency Formation (Fodor, 1978; Stowe, Tanenhaus, & Carlson, 1991). There is evidence for such a process across languages and experimental techniques (Hawkins, 1999; Phillips & Wagers, 2012). Thus, in (25b), speakers have to parse several words and embedding levels (*did you say* [*Mary wants me* [*to*) before understanding that *which book* is the object of *read*.

(25)
  a. I read [a book] yesterday
  b. [Which book] did you say Mary wants me to read?

### 1.6 Gap‐mediated versus immediate dependency formation

Two main syntactic accounts of this Active Dependency Formation have been given: gap‐mediated dependency formation and immediate dependency formation (Traxler & Pickering, 1996). According to the gap‐mediated dependency formation hypothesis, speakers might be searching for gaps related to a filler (Chow & Zhou, 2019; Fodor, 1978). This hypothesis is supported by evidence of filler‐reactivation at the gap location, which has been found across languages, in English (Gibson & Warren, 2004; Keine, 2020; Nicol & Swinney, 1989; Nicol, Fodor, & Swinney, 1994), in German (Clahsen & Featherston, 1999), or in Japanese (Nakano, Felser, & Clahsen, 2002). Several of these studies used a cross‐modal lexical priming paradigm, where participants listened to sentences presented to them over headphones and then had to perform a lexical decision task at what seemed like an arbitrary point during the sentence (Nicol & Swinney, 1989; Nicol et al., 1994). The researchers found faster lexical decision at the gap site for words related to the filler compared to unrelated words. For example, participants might be asked to listen to a sentence like (26).

(26) The old man picked up the apple which the baby in the carriage threw *e* in the gutter.

Then, at the point where they just heard *threw*, they would be asked whether fruit or bench is a word or not. Note that the filler *which* refers to *apple*, and is related to fruit but not bench. Nicol and Swinney (1989) found faster response times for fruit relative to bench, and concluded that an empty element at *threw* (coindexed with *apple*) led to this speed‐up in reaction time.

But McKoon & Ratcliff (1994); McKoon, Allbritton, & Ratcliff (1996) observed a confound in the interpretation of the effects: words like fruit fit better following a verb like *threw*, independently of whether there is a filler like *which* before, because a fruit can be thrown but a bench cannot be thrown. McKoon, Allbritton, & Ratcliff (1996) reran the experiment with items where the control materials were matched for this context effect, and they found no effect of filler. Although there is some discussion of this confound in later English experiments (Love & Swinney, 1996; Nicol, et al., 2006) (without the issue being fully resolved), studies in other languages that reported evidence of empty elements (such as Clahsen & Featherston, 1999 in German) also have this contextual confound. Yet, another line of research supporting the gap‐mediated dependency formation comes from studies about pre‐verbal gap processing. For instance, Lee (2004) argued for gap‐mediated dependency formation in English, based on the evidence that speakers can associate a filler with its pre‐verbal gap before reaching the verb position (but see Omaki et al. (2015); Aoshima, Phillips, and Weinberg (2004) for a discussion on pre‐verbal gap‐filling in Japanese and English).

Some data that have been taken as evidence to support empty categories (and transformational accounts) do not actually distinguish transformational from nontransformational analyses of the same sentences. For example, the materials in papers by Gibson & Warren (2004); Keine (2020) involved intermediate positions in long‐distance dependencies, as in (27a) and (27b):

(27)
  a. The manager who 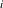 the consultant claimed $e_{i}$ that the new proposal had pleased $e_{i}$ will hire five workers tomorrow.
  b. The manager who 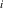 the consultant's claim about the new proposal had pleased $e_{i}$ will hire five workers tomorrow.

The hypothesis was that the connections in (27a) are more local than in (27b) because of an intermediate empty element in (27a) (the $e_{i}$ before *that* in (27a)). These researchers found faster self‐paced reading times the region initiated with the verb *had pleased* in (27a) versus (27b). This provided existence consistent with the existence of an empty element in materials like (27a) relative to materials like (27b).

While the data from these papers are consistent with this hypothesis, the data are also consistent with the hypothesis that there is just intermediate feature‐passing in the materials under investigation, as proposed by theories such as Head‐driven Phrase Structure Grammar (HPSG) (Sag, Wasow, & Bender, 1999) and Lexical‐Functional Grammar (LFG) (DalrympleLowe, & Mycock, 2019, p. 689‐693). Gibson & Warren (2004); Keine (2020) did not design their materials to distinguish empty categories from feature passing. All modern theories have one of these variants, so these studies do not actually provide evidence for intermediate empty categories. They simply suggest the existence of intermediate activation, which could be explained by empty categories, feature passing, or something else.

In contrast to the Gap‐mediated Hypothesis, several studies have provided evidence for an Immediate Dependency Formation Hypothesis, or Direct Association Hypothesis (Pickering and Barry, 1991; Pickering, 1994, 1993). Under this hypothesis, dependencies with fillers are made before the gap location is actually found, as soon as enough information is provided to interpret the filler, for example, when finding the head it depends on, or even earlier (Omaki et al., 2015).

Pickering & Barry (1991) provided contrasts like those in (28) from examples (32) and (33) in Pickering & Barry (1991, p. 237). Sentence (28a) seems less acceptable than (28b), yet, these two sentences receive similar structures under a transformational analysis, since the empty category e in the extracted version (28b) is in the same position as the phrase *a prize* in the nonextracted version (in 28b, the phrase *which* is assumed to leave this empty category). Pickering & Barry (1991) suggest that (28b) is less complex because the filler *which* can be directly associated with its head *gave*, whereas in (28a), speakers have to process the 34‐word long NP *every student… military rulers* before finding the NP *a prize*, leading to processing difficulty when they have to connect it to *gave*. The authors conclude that direct association explains the intuitive contrast, whereas a theory with empty elements does not.

(28)
  a. ↓ We gave every student capable of answering every single tricky question on the details of the new and extremely complicated theory about the causes of political instability in small nations with a history of military rulers [a prize]^5^
  b. That's the prize which we gave every student capable of answering every single tricky question on the details of the new and extremely complicated theory about the causes of political instability in small nations with a history of military rulers *e*

The implicit idea underlying Pickering & Barry (1991)'s observation is that longer dependency distance between words in a sentence are associated with greater processing cost. This is the explicit idea in the Dependency Locality Theory, discussed below.

Traxler and Pickering (1996, Experiment 1) provide evidence for Immediate Dependency Formation using eye‐tracking data in reading. Contrasting plausible and implausible filler‐verb combinations (like *That's the garage/pistol in/with which the heartless killer shot the man yesterday afternoon*), they showed that reading difficulty occurs on the head‐verb (here, *shot*) rather than on the hypothesized gap location (after *man*).

### 1.7 Dependency Locality Theory

According to the Dependency Locality Theory (DLT) (Gibson, 1998, 2000), there is an *integration cost* associated with connecting words that need to be interpreted together in the structure of a sentence. The integration costs are approximately given by the lengths of the dependency arcs (see also Futrell, Gibson, & Levy, 2020 for a related idea, information locality, which leads to similar predictions). To take a concrete example, we can compare the two sentences in (29). Intuitively, sentence (29b) is less acceptable, and this can be attributed to the greater dependency distance going from the head *gave* to the dependent *a book*. The DLT thus establishes a correlation between dependency distance and integration cost: the longer the dependency between two words, the higher the integration cost of the later word, which causes acceptability reductions. Evidence for the DLT hypothesis is provided by reading times in English (Bartek, Lewis, Vasishth, & Smith, 2011; Grodner & Gibson, 2005) and brain activation in the language cortex when listening to stories (Shain, Blank, Fedorenko, Gibson, & Schuler, 2022). It also correctly predicts the short‐before‐long preference observed in head‐initial languages, the long‐before‐short preference in head‐final languages (Hawkins, 2014), and the tendency for dependency distance minimization across languages (Futrell, Levy, & Gibson, 2020; Gildea & Temperley, 2007; Liu, 2020).

(29)
  a. I gave the student [a book]
  b. ↓ I gave the student who was able to answer all the questions [a book]

The sentences in (29) can be represented by the dependency structures in (30), using Dependency Grammar (De Marneffe & Nivre, 2019; Gibson, 2025; Hudson, 2015; Mel'cuk, 1988; Osborne, 2014; Tesnière, 1959). This framework assumes that sentence structure can be represented by Directed Acyclic Graphs (i.e., trees), where nodes are words and edges are dependencies. We draw dependencies from heads to dependents and we only draw arcs relevant to the current discussion. When we will compare the no‐empty‐categories (no‐ECs) and the with‐empty‐categories (+ECs) analyses, we will draw filler‐head dependencies in red above the sentence and filler‐gap dependencies in blue below the sentence. We provide dependency distances on the arc in terms of the number of words between the start and end of the arc (more precisely, taking the difference between the head and dependent position indices, Liu, 2008). In the DLT, dependency distance is computed based on the number of new discourse referents: nouns (not pronouns) and tensed verbs. Computing distance in terms of words versus discourse referents has no bearing on the observations made by Pickering & Barry (1991), so we use the simpler word‐based distance metric here for exposition purposes (cf. Hawkins, 1994, 2004).

 
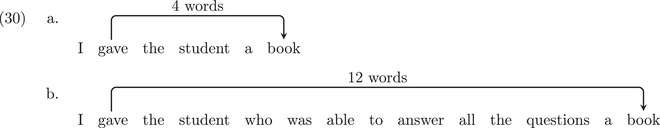


### 1.8 No‐ECs versus +ECs analyses

The DLT is relevant for our purposes when it comes to filler‐gap constructions such as (31a). The blue analysis in (31b) is a +ECs analysis. Here, the filler connects to an EC following *book*. The no‐ECs analysis is shown in red, where the filler connects to its role‐assigning head *give* directly. As can be seen in the diagram, the filler‐gap distance in blue is longer (seven‐words long) than the filler‐head distance in red (four‐words long).

 
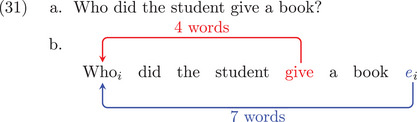


Applying dependency length complexity to +ECs and no‐ECs structures, we can evaluate the predictions of the respective theories regarding processing costs. In this paper, we evaluate two contrasts used by Pickering & Barry (1991) to address the presence of empty categories in syntactic structures. The first contrast, tested in Experiment 1, deals with nested filler‐gap constructions using NP and PP fillers. In this first case, we will compare sentences with the same filler‐gap distance but varying filler‐head distances. The second contrast is based on ditransitive verbs used in *wh‐*interrogative sentences (Experiment 2). In this second case, we will test the opposite comparison: the same filler‐head distance with varying filler‐gap distances. The first contrast is found in Pickering & Barry (1991, p. 240).

(32)
  a. This is the saucer [on which] Mary put the cup [into which] I poured the tea
  b. ↓ This is the saucer [which] Mary put the cup [which] I poured the tea into on

In (32b), the two filler‐gap constructions are nested within each other: *which I poured the tea into* is contained in the larger phrase *which Mary put the cup which I poured the tea into on*. It has long been known that structures with nested dependencies are difficult to produce and comprehend, for example, Yngve (1960); Chomsky and Miller (1963); Gibson (1991); Lewis (1996); Gibson (1998, 2000); Lewis and Vasishth (2005). For example, the nested relative clause example in (33b) has a similar meaning as the non‐nested relative clause example in (33a), in terms of the semantic relationships among the words, but (33b) is much more difficult to understand:

(33)
  a. The cat chased the dog which bit the boy who was upset.
  b. ↓ The boy who the dog which the cat chased bit was upset.

The dependency structures for (33a–33b) are provided in (34a–34b). Dependency length complexity explains the complexity of nested structures in terms of long‐distance dependencies: nested structures contain multiple long‐distance dependencies, whereas the control structures—which mean close to the same thing—involve shorter dependencies. For example, the *boy‐was* dependency in (33b) is nine‐words long, while it is only two‐words long in (33a).

 
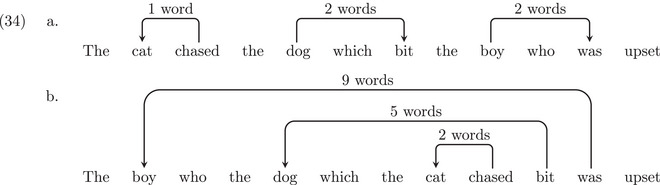


If we analyze sentence (32b) with dependency arcs (35), we can see that both the +ECs analysis (in blue) and the no‐ECs analysis (in red) involve nested dependencies. As a consequence, they both predict the kind of acceptability reduction we typically find with nested structures, as in (34b).

 
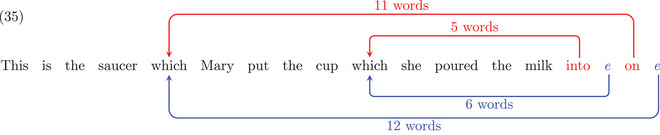


However, differences appear when we consider sentence (32a). For the +ECs analysis, dependencies remain nested as shown in (36). The arcs connecting the fillers with their corresponding empty category are as long as in the structure (35): 13 words and 6 words for the first and the second filler, respectively. This may be surprising given that (32a) seems intuitively more acceptable than (32b). Under a dependency length complexity approach, this acceptability difference between the two sentences would not be explained if the structures make use of ECs. By contrast, if we adopt a no‐ECs analysis, the dependency distances decrease compared to (35): from 11 words and 5 words to 3 words for each dependency. Thus, in a no‐ECs analysis, sentence (32a) does not involve a nested structure. The dependency distances are shorter, and the sentence would be predicted to be more acceptable.

 
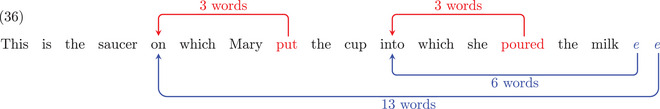


But these differences remain to be tested experimentally, in order to provide evidence for either theory. This is the purpose of Experiment 1.

## 2 Experiment 1

### 2.1 Materials

Our goal is to investigate the effect of long nested dependencies on acceptability comparing structures with (+ECs) and without ECs (no‐ECs). We started with the contrasting pairs used in Pickering & Barry (1991) discussed in (32) above. In a first experiment (Experiment A in the Appendix), we tested the complexity of such sentences compared to two simpler baseline conditions: a sentence coordination (37a) and a simple relative clause (37b).

(37)
  a. Mary put the cup on the saucer and she poured the milk into the cup.
  b. Relative clause: Mary poured the milk into the cup which she put on the saucer.

We found that nested relative clauses with two NP fillers (32a) are less acceptable than with two PP fillers (32b). Reviewers pointed out that this experiment did not include appropriate manipulation of the category of the filler to vary dependency distance because they were both changed at the same time (two filler NPs vs. two filler PPs). We agree with the reviewers; in order to keep the presentation brief, we moved the presentation of this experiment to the Appendix.

To overcome the limitation observed by the reviewers, we ran Experiment 1 with conditions with intermediate cases of difficulty, in order to isolate the effect of dependency length, as shown in (38). We used a 2×2 design, manipulating dependency distance in two nested filler‐gap constructions. The first filler is either a PP (38a–38b) or an NP (38c–38d). The second filler is also either a PP (38a–38c) or an NP (38b–38d).

(38)
  a. PP‐filler1; PP‐filler2: This is the saucer [on which] Mary put the cup [into which] she poured the milk.
  b. PP‐filler1; NP‐filler2: This is the saucer [on which] Mary put the cup [which] she poured the milk into.
  c. NP‐filler1; PP‐filler2: This is the saucer [which] Mary put the cup [into which] she poured the milk on.
  d. NP‐filler1; NP‐filler2: This is the saucer [which] Mary put the cup [which] she poured the milk into on.

Under a no‐ECs analysis of these conditions, the filler NPs involve longer dependencies than the filler PPs. Under the DLT, the longer a dependency is, the lower the acceptability. So, the four conditions in this manipulation are predicted to yield an acceptability continuum under a no‐ECs analysis: PP fillers correspond to the shortest dependencies, with the consequence that the PP‐filler1‐PP‐filler2 condition (38a) should be the most acceptable, and the NP‐filler1‐NP‐filler2 condition (38d) should be the least acceptable. The PP‐filler1‐NP‐filler2 and NP‐filler1‐PP‐filler2 conditions represent intermediate cases where the no‐ECs analysis predicts that the NP‐filler1‐PP‐filler2 condition (38c) would be rated lower than the PP‐filler1‐NP‐filler2 condition (38b) because the NP filler dependency is longer in the former (*which… on*) than in the latter (*which… into*). Let $R_{c}$ be the mean acceptability rating in a given condition c. Thus, a no‐ECs analysis predicts the following pattern of acceptability, from the most acceptable structure the least acceptable one: $R_{PP-PP}>R_{PP-NP}>R_{NP-PP}>R_{NP-NP}$. In contrast, a +ECs analysis predicts no acceptability differences among these conditions, because dependency distances are kept constant, with empty categories located at the end of the sentence in all conditions.

We created 20 items following the structure in (38). Proper names were controlled for gender with as many typically female names (*Joan, Jane, Emily, Emma*…) as typically male names (*John, Thomas, William, Peter*…). All common nouns were inanimate (*supermarket, well, bucket, pot, bottle*…) referring mainly to places and containers. Most verbs were causative movement verbs (*put, bring, plant, spill, cover*…) requiring a PP‐locative complement, introduced by a preposition (*on, onto, in, into, under, at*). All items can be found on the OSF project of this study (https://osf.io/dyfqh/). These items were put together with 44 fillers containing other types of structures (simple declarative clauses, interrogative and exclamative clauses). Two practice items were also included at the beginning of the task. The experiment thus included 66 trials in total.

### 2.2 Procedure

The task was an acceptability judgment task on the Ibex Farm platform (Drummond, 2013). We used the Prolific platform to recruit 40 native English speakers located in the United States. Participation was anonymous, voluntary, and paid ($4). All participants gave their consent for the data analysis, and they can retract their data using their Prolific ID.

Participants read sentences on their screen and gave a rating on a 1–7 Likert scale (“unacceptable” to “acceptable”). Each sentence was displayed one at a time. All sentences were followed by a yes/no comprehension question. At the beginning of the experiment, participants had to fill out a short sociolinguistic questionnaire (native language(s), country of L1 acquisition, age, gender, other known languages).

The presentation order of the items and fillers was randomized and the presentation of the four conditions was counterbalanced across four lists of items following the Latin Square design, each item being presented in only one of the four conditions in each list.

### 2.3 Participants

We excluded one participant who got less than 80% correct responses on comprehension questions, namely, 78%. The average accuracy score for the remaining participants is 95% (*SD* = 0.05). We thus analyzed data for 39 participants: 21 women and 18 men. Mean age is 44 years (*SD* = 14.9, Max = 73 years, Min = 23 years).

### 2.4 Data analysis

We analyzed acceptability judgment data with a cumulative ordinal regression model using the *logit* link function (Bürkner & Vuorre, 2019). We ran the model on R using the Bayesian framework (package *brms*, Bürkner, 2017). All data and scripts can be found on the OSF project of this study (https://osf.io/dyfqh/). The posterior predictive checks of the models we use are also given in these Supplementary Files.

We fitted a model to acceptability rating according to the category of the two fillers (filler1, filler2), which include two levels: PP versus NP. We used “PP” as the reference level for each predictor. We used a centered treatment coding (−0.5, 0.5) so that the “PP” level is coded as −0.5, and the “NP” level as 0.5. We included by‐participant and by‐item intercepts and slopes as random effects. We used weak informative priors for fixed‐effects coefficients (Normal distribution with μ=1;σ=0) and used the default priors for the thresholds and the standard deviation of random effects (Student's *t*‐distribution with μ=0;σ=2.5;ν=3).

Predictors of ratings are the categories of the two fillers and their interaction. The model's parameters are thus the following: $\beta_{\mathrm{NPFiller}1}$, $\beta_{\mathrm{NPFiller}2}$, and $\beta_{\mathrm{NPFiller}1:\mathrm{NPFiller}2}$, and the model formula is show in (39). As we have no hypothesis about group‐level correlations between predictors, we did not include them.

(39) rating ∽ category of filler1 * category of filler2 +
(category of filler1 * category of filler2 ‖ participant) +
(category of filler1 * category of filler2 ‖ item)

We test for reliability of the differences investigated considering there is compelling evidence for an effect if the probability *p* of the posterior distribution β differing from zero is greater than 0.95, that is, either *p* (β>0)≥.95 or *p* (β<0)≥.95.

### 2.5 Results

**Fig. 1:**
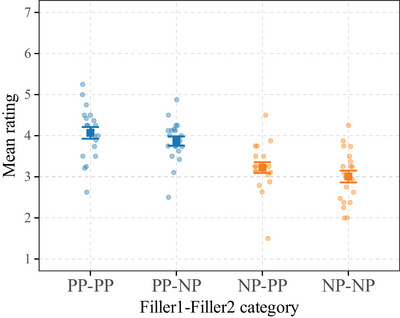
Mean ratings by item (dots) and by condition (squares). Participants = 39, items = 20, observations = 780. Error bars represent standard error.

We report mean ratings for each condition in Fig. 1. Table 1 summarizes the characteristics of the posterior distributions of the ordinal model. We use E(·) as estimates of the model.

**Table 1:** Summary of the population‐level posterior distributions of the ordinal model

Parameter	Estimate	Standard deviation	95% Credible interval
**Thresholds**			
$\tau_{1}$	−2.72	0.40	[−3.53; −1.96]
$\tau_{2}$	−0.89	0.38	[−1.65; −0.15]
$\tau_{3}$	0.20	0.38	[−0.57; 0.94]
$\tau_{4}$	1.24	0.38	[0.49; 1.98]
$\tau_{5}$	2.72	0.39	[1.96; 3.48]
$\tau_{6}$	3.99	0.41	[3.19; 4.82]
**Predictors**			
$\beta_{\mathrm{NPFiller}1}$	−1.40	0.26	[−1.91; −0.89]
$\beta_{\mathrm{NPFiller}2}$	−0.27	0.17	[−0.6; 0.07]
$\beta_{\mathrm{NPFiller}1:\mathrm{NPFiller}2}$	−0.11	0.33	[−0.78; 0.52]

*Note*. By‐item and by‐participant random intercepts and slopes. Reference level of the predictors is set to “PP” for both fillers. Negative estimates for predictors correspond to an acceptability decrease.

The structure involving two filler PPs (PP‐filler1‐PP‐filler2) received the highest rating ($R_{PP-PP}=4.07$). When the second filler is an NP (PP‐filler1‐NP‐filler2), the mean rating decreased slightly ($R_{PP-NP}=3.87$). When the first filler is an NP (NP‐filler1‐PP‐filler2), the mean rating decrease was more important ($R_{NP-PP}=3.22$). Finally, the structure with two NP fillers (NP‐filler1‐NP‐filler2) received the lowest rating ($R_{NP-NP}=3.01$). We thus get the following acceptability pattern: $R_{PP-PP}>R_{PP-NP}>R_{NP-PP}>R_{NP-NP}$.

Our data provide compelling evidence for a main effect of the category of filler 1: when filler is an NP, compared to a PP, acceptability decreases (E($\beta_{\mathrm{NPFiller}1}$) = −1.4; *SD* = 0.26; 95% CrI = [−1.91,−0.89]; *p*($\beta_{\mathrm{NPFiller}1}<0$) = 1). We report weaker evidence for a main effect of the category of filler 2 (E($\beta_{\mathrm{NPFiller}2}$) = −0.27; *SD* = 0.17; 95% CrI = [−0.6, 0.07]; *p*($\beta_{\mathrm{NPFiller}2}<0$) = .94). We also provide compelling evidence that the main effect of the category of filler 1 is stronger than the main effect of the category of filler 2: having filler 1 as an NP decreases the acceptability more than having filler 2 as an NP (E($\beta_{\mathrm{NPFiller}1}-\beta_{\mathrm{NPFiller}2}$) = −1.13; *SD* = 0.31; 95% CrI = [−1.74,−0.53]; *p*($\beta_{\mathrm{NPFiller}1}-\beta_{\mathrm{NPFiller}2}<0$) = 1). Finally, there was no evidence for an interaction between the category of the two NPs (E($\beta_{\mathrm{NPFiller}1:\mathrm{NPFiller}2}$) = −0.11; *SD* = 0.33; 95% CrI = [−0.78, 0.52]; *p*($\beta_{\mathrm{NPFiller}1:\mathrm{NPFiller}2}<0$) = .64), 

### 2.6 Discussion

The results of Experiment 1 are consistent with those from preliminary Experiment A, where we found that filler‐head distance affects acceptability, not filler‐gap distance. In particular, Experiment 1 showed that acceptability is sensitive to the category of the fillers involved in nested filler‐gap constructions. This sensitivity contradicts the prediction of the +ECs theory. Under a +ECs analysis, filler‐gap distances are constant across filler categories, since the empty categories are located at the end of the sentence, in the canonical position of the filler phrase. This incorrectly predicts that all sentences should receive the same acceptability. In contrast, the predictions of dependency length complexity under a no‐ECs analysis ($R_{PP-PP}>R_{PP-NP}>R_{NP-PP}>R_{NP-NP}$) are supported by our data: when a filler is an NP in these materials—so that it requires a longer filler‐head distance—sentence acceptability is lower.

Moreover, we found that the acceptability reduction triggered when the first filler is an NP is bigger than when the second filler is an NP. The dependency length complexity with no ECs predicts this difference, as the first filler is more distant from its head than the second filler when they are NPs. This shows that the longer a dependency is, the more it reduces acceptability. This experiment thus supports the no‐ECs approach. Indeed, the results suggest that there is nothing special about the double‐nesting with NP fillers: their unacceptability is due to the cumulative complexity of each long‐distance dependency (filler1 and filler2), and there is no extra acceptability reduction associated with their combination (no interaction).

The category manipulation in the current experiment—PP versus NP —also modifies the construction type: fronted PP fillers versus preposition‐stranding for NP fillers. These constructions have other differences in addition to dependency distance. In particular, preposition‐stranding is generally considered to be more complex than having a PP filler, even if it can also provide some advantages, such as a facilitated phrase edge recognition (Enzinna, 2013; Günther, 2021; Klein, 1993). There are two factors that contribute to the complexity of preposition‐stranding. First, as observed here, preposition‐stranding requires connecting a filler with a more distant head, a preposition, whereas PP‐fillers involve shorter dependencies between the filler and the verb head.

Second, preposition‐stranding is complex because fillers in this construction are NPs, which make them potential dependents of many words, including verbs and prepositions, which may lead to temporary ambiguity and may be difficult for the comprehender, as in (40) (Crain & Fodor, 1985; Stowe, 1986; Traxler and Pickering, 1996).

(40)
  a. We like the book [which] the author wrote with great dedication **about** (adapted taken from Traxler and Pickering, 1996, 465)
  b. The book which I wrote **a comment** on.

In these sentences, the NP filler *which* can be first analyzed as the object of the verb *wrote* (*I wrote a book*). However, this first analysis has to be revised when *about* is encountered in (40a), because this preposition requires a complement. In (40b), something similar happens when *a comment* is encountered, because this phrase is the actual object of *wrote*, and *which* has then to be attached later, to the preposition *on*. More specifically, this last garden‐path effect is called a filled‐gap effect (Crain & Fodor, 1985; Stowe, 1986), because it is triggered when the expected gap after the verb (here, *wrote*) appears to be filled (here, by *a comment*).

In our experiment, it is possible that some preposition‐stranding constructions may have triggered filled‐gap effects. It is then a question as to whether the distance‐based complexity effects that we observed here might be explained in terms of filled‐gap effects in the NP‐filler items.

Examples in (41) illustrate cases where a filled‐gap effect can occur at the bold phrases. In these examples, when encountering the verbs *put* or *forgot*, the parser may analyze the filler *which* as its object. Then, when encountering a subsequent NP, *the cup* or *his bag*, the actual object is found, showing that the first analysis was wrong.

(41)
  a. This is the saucer [which] Mary put **the cup** into which she poured the milk on.
  b. This is the room into which John brought a chair [which] he forgot **his bag** on.

There are two reasons to think that our observed effects are not filled‐gap effects in the NP‐filler examples. First, if filled‐gap effects are responsible for the observed pattern of acceptability, we would expect them to have the same effect size with the first filler and the second filler. Crucially, we showed that preposition‐stranding with the first filler (as in 41a) incurs a bigger acceptability reduction than preposition‐stranding with the second filler (as in 41b). As filled‐gap effects occur in both cases, the observed difference has to be related to another factor. Under a no‐ECs analysis, the only difference between the stranding with the first filler and the second filler is the filler‐head distance: it is longer for the first filler, so it should be less acceptable. Again, the filler‐gap distance assumed in the +ECs analysis is kept constant regardless of the constructions (preposition‐stranding vs. PP‐fillers), so we would expect either no difference between them (ignoring the potential filled‐gap effects) or the same difference for both the first and second fillers (considering the potential filled‐gap effects). None of these predictions are borne out.

Second, our items vary in regard to the presence of potential filled‐gap effects when fillers are NPs. While the examples in (41) contained likely filled‐gap effects, the examples in (42) do not. Indeed, *to write a desk* or *to impress a show* are not plausible events, making the analysis of the NP fillers as objects less likely, and the presence of the subsequent object NP less surprising^6^ (Crain & Fodor, 1985; Stowe, 1986; Traxler and Pickering, 1996).

(42)
  a. This is the desk [which] Bob wrote the book into which he put a bookmark on.
  b. This is the stage on which Lia performed the show [which] she impressed the audience with.

If filled‐gap effects played a systematic role in our results, we would expect the sentences in (42) to be more acceptable than sentences in (41), compared to their respective PP‐filler counterparts.

**Fig. 2:**
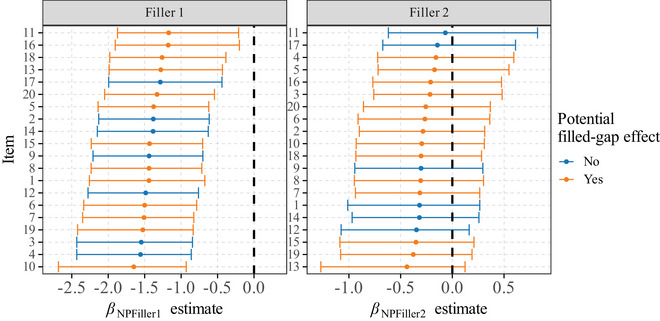
Mean estimate of the $\beta_{\mathrm{NPFiller}}1$ posterior across items. Error bars represent 95% CrI.

To evaluate this prediction, we analyzed the by‐item random effects of our model. This allows us to assess the item variation in the effect sizes of having NP fillers compared to PP fillers. Out of our 20 items, the NP filler is an unlikely object for the first verb in seven cases, and for the second verb in six cases (according to both authors' intuitions). We expect those cases to exhibit smaller effect sizes, that is, their NP condition is more acceptable. Fig. 2 shows the means and 95% credible intervals of the posterior distribution $\beta_{\mathrm{NPFiller}1}$ and $\beta_{\mathrm{NPFiller}2}$ for each item.

The left panel of Fig. 2 shows that the acceptability reduction effect triggered by the category of the first filler (NP vs. PP) is robust to item variation and cannot be associated with the presence or absence of potential filled‐gap effects in a systematic way. We find that items with no potential filled‐gap effect are distributed among the other items in a continuous manner. Even if some items may suffer filled‐gap effects, there is still a reliable effect when they are absent (E($\beta_{\mathrm{NPFiller}1}$) ∈ [−1.56;−1.29], *p*($\beta_{\mathrm{NPFiller}1}<0$) ∈ [0.99; 1]), which can be, in those cases, attributed to dependency distance. The right panel shows that there is also variation among items in the effect of the second filler. Overall, this effect is weaker, which is not predicted if only filled‐gap effects are responsible for the acceptability reduction. Items without potential filled‐gap effects can have relatively weak effects (items 11 and 6), as well as stronger effects (items 1, 14, and 12). Items with potential filled‐gap effects are again distributed along the scale with no clustering. The role of potential filled‐gap effects seems thus marginal in explaining the variation across items.

Finally, preposition‐stranding can also be less acceptable for prescriptive reasons,^7^ as it is criticized by purists and as having a PP‐filler is generally considered more formal (Yáñez‐Bouza, 2008). However, given the complexity of our items, evidenced by their overall low acceptability, even with PP fillers (see FIgure 1), we think that prescriptivism is unlikely to be a major factor for the observed differences.

## 3 Experiment 2

In Experiment 1, we compared cases where the filler‐head distance varies, with the longer distances being less acceptable, whereas filler‐gap distances were kept identical across conditions. Consequently, the dependency length complexity approach with ECs predicted a null effect. To better assess our hypotheses, Experiment 2 compares cases where the filler‐gap distances vary. In such cases, the +ECs analysis predicts different dependency distances, which would lead to acceptability differences. By contrast, in the no‐ECs analysis, the distances would be the same. Thus, the dependency length complexity approach would predict no acceptability difference under a no‐ECs analysis.

To test this idea, we investigated materials similar to the contrast suggested by Pickering & Barry (1991, p. 236–237), exemplified in (43), which compares the position of the complements of ditransitive verbs in *wh‐*interrogative constructions. Ditransitive verbs exhibit a specific argument structure with two object NPs (the double‐object construction), where the first one is a recipient and the second one is a theme. This makes the presence of a theme NP highly predictable when a recipient NP has been found.

(43)
  a. [Which prize] did Mary give the students after they answered the question correctly without getting any help?
  b. [Which prize] did Mary give the students who could answer the question correctly without getting any help?

The blue dependency arcs displayed in (44) indicate the +ECs analysis. We abbreviate the long modifiers *correctly without getting any help* as an ellipsis “…”. As shown in (44a), the empty category for *which prize* is located before the adjunct clause (*after they answered the question*), which makes the dependency distance short (six‐words long). But, when this adjunct clause is turned into a relative clause (44b), with the same content modifying the noun *student*, the empty category has to be placed at the end of the sentence, making the dependency distance 16‐words long. The dependency length complexity approach predicts that this dependency distance increase from (44a) to (44b) triggers an acceptability reduction. Red arcs show an analysis with no empty category. This time, the structure of the clausal modifier does not affect the filler‐head distance linking the filler *which prize* with its head *give* at the beginning of the sentence: it is three‐words long in both cases.

 
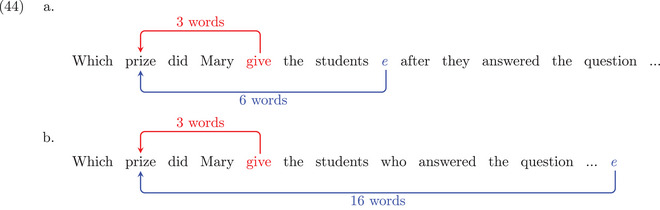


As a consequence, comparing these two sentences now leads to opposite predictions compared to Experiment 1: the dependency length complexity approach predicts a difference between (44a) and (44b) if the underlying structures contain empty categories, whereas no difference is expected if there are no ECs.

If we take the declarative version of sentences (44), given in (45), we can control for the effect of dependency distance in non‐filler‐gap constructions. Regardless of the assumption about empty categories, sentence (45b) involves a longer dependency distance than (45a) and is thus expected to be less acceptable. The difference found in (45) should be similar to the one found in (44) if we adopt +ECs structures. However, when there are no EC structures, we predict a difference in (45) that should be absent in (44). Experiment 2 investigates these predictions.

 
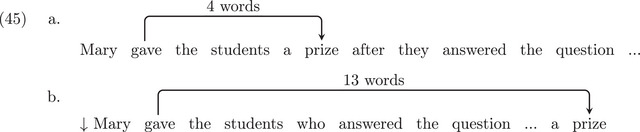


### 3.1 Materials

In this experiment, we tested sets of materials like those introduced in (43). We manipulated two variables: construction, with two levels (“declarative” and “interrogative”) and np object length, with two levels (“short” and “long”), to which we add a third baseline condition, in order to isolate the declarative versus interrogative effect. This corresponds to a 2x3 design, as illustrated in (46–48). Fillers and their canonical counterparts are enclosed in squared brackets.

(46) Baseline:
  a. Declarative: Mary gave the students [a prize].
  b. Interrogative: [Which prize] did Mary give the students *e*?
(47) Short NP object
  a. Declarative: Mary gave the students [a prize] after they answered the question correctly without getting any help.
  b. Interrogative: [Which prize] did Mary give the students *e* after they answered the question correctly without getting any help?
(48) Long NP object
  a. Declarative: Mary gave the students who could answer the question correctly without getting any help [a prize].
  b. Interrogative: [Which prize] did Mary give the students who could answer the question correctly without getting any help *e*?

These materials were designed to compare filler‐head and filler‐gap distances. The dependency length complexity approach makes different predictions for theories with and without ECs in examples (47) and (48). With +ECs, the filler‐gap distance in (48b) is greater than in (47b). This would imply that sentence (48b) with a long filler‐gap distance is less acceptable than sentence (47b). This difference should be parallel to the one found in the declarative counterparts of these sentences, namely, (47a) and (48a). Sentence (48a) presents a longer distance dependency (*give*… *a prize*) than (47a) and should thus be less acceptable too. With no‐ECs, the declarative sentences in (47a) and (48a) should still show an acceptability difference, given the increase of the dependency distance in (48a). However, this difference is predicted to disappear in the interrogative versions, because there the filler‐head distance (*which prize*… *give*) is kept constant.

Theories with +ECs or no‐ECs structures, therefore, make distinct predictions with respect to these materials. In both kinds of theories, we expect an acceptability difference between short and long NP objects for declarative sentences, because these materials do not involve filler‐gap dependencies. For interrogative sentences, the +ECs theories predict the same acceptability difference found in declaratives, whereas the no‐ECs theories predict no differences in the interrogatives.

We created 24 items using the six conditions exemplified in (46–48). We used two classes of ditransitive verbs: 15 giving verbs (*give, offer, send, sell*, etc.) and nine communication verbs (*teach, tell, read, show*, etc.). Proper names included 14 typically female names (*Emma, Jane, Emily*, etc.) and 10 typically male names (*Peter, Liam, Henry*, etc.). The relative clause in the long‐condition and the adjunct clause in the short‐dependency always have the same content, and then approximately matched in length. The only difference was in the introductory words of the clauses: *who* for relative clauses, *conjunction* + *they* for adjunct clauses. We used the same 44 fillers as in Experiment 1 with the same two training items, leading to 70 trials in this experiment. All materials can found found in the OSF project of this study (https://osf.io/dyfqh/). After running the experiment, we found a typographical error in the interrogative conditions of item 18. We thus removed this item from the analysis.

### 3.2 Procedure

We used the same procedure as in Experiment 1. We recruited 41 native English speakers located in the United States using the Prolific platform. Participants completed an acceptability judgment task. They read sentences on the screen and rated them on a 1–7 Likert scale (“unacceptable” to “acceptable”). All sentences were followed by a yes/no comprehension question. At the beginning of the experiment, participants had to fill out a short sociolinguistic questionnaire (native language(s), country of L1 acquisition, age, gender, other known languages). Participation was anonymous, voluntary, and paid ($4). All participants gave their consent for the data analysis, and they can retract their data using their Prolific ID.

As in Experiment 1, we randomized the presentation order for critical items and fillers. The presentation of critical items followed a Latin Square: they were assigned to six lists so that each item appeared only once in one condition in each list.

### 3.3 Participants

One participant scored 70% on the comprehension questions, which is lower than our 80% threshold. So, we removed this participant and then analyzed the data for 40 participants: 18 men and 22 women (mean age = 43.2 years, *SD* = 14.9, Max = 77 years, Min = 20 years). Their average accuracy score to comprehension question was 98% (*SD* = 0.02).

### 3.4 Data analysis

To analyze acceptability ratings, we used the same kind of model as in Experiment 1: a cumulative ordinal regression with the *logit* link function run on R (Bürkner, 2017; Bürkner & Vuorre, 2019).

The model is fitted to acceptability ratings with two predictors: construction and dependency distance. Construction has two levels with “declarative” taken as the reference level, and NP object length is included as a categorical variable with three levels (baseline, short, long), with “short” as the reference level. We used a centered treatment coding for the predictors: 0 versus 1 contrasts are created and then centered around zero. We included a full random effect structure, with by‐participant and by‐item intercepts and slopes. We used weak informative priors for fixed‐effects coefficients (Normal distribution with μ=1;σ=0) and used the default priors for the thresholds and the standard deviation of random effects (Student's *t*‐distribution with μ=0;σ=2.5;ν=3). The model formula is given in (49).

(49) rating ∽ construction * np object length +
(construction * np object length ‖ participant) +
(construction * np object length ‖ item)

### 3.5 Results

**Fig. 3:**
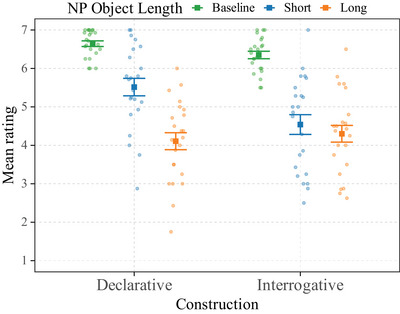
Mean ratings by item (dots) and by condition (squares). Participants = 40, items = 23, observations = 920. Error bars represent standard error.

Mean acceptability ratings are shown in Fig. 3, and parameter estimations of the corresponding ordinal model are shown in Table 2.

**Table 2:** Summary of the population‐level posterior distributions of the ordinal model

Parameter	Estimate	Standard deviation	95% Credible interval
**Thresholds**			
$\tau_{1}$	−5.28	0.42	[−6.13;−4.45]
$\tau_{2}$	−3.77	0.39	[−4.54; −3.01]
$\tau_{3}$	−2.60	0.37	[−3.34; −1.87]
$\tau_{4}$	−1.76	0.37	[−2.49; −1.04]
$\tau_{5}$	−0.72	0.36	[−1.43; −0.01]
$\tau_{6}$	0.93	0.36	[0.22; 1.65]
**Predictors**			
$\beta_{\mathrm{Interrogative}}$	−0.73	0.22	[−1.17; −0.29]
$\beta_{\mathrm{Baseline}}$	2.80	0.43	[1.94; 3.63]
$\beta_{\mathrm{LongNP}}$	−1.02	0.30	[−1.6; −0.42]
$\beta_{\mathrm{Interrogative}:\mathrm{Baseline}}$	0.21	0.46	[−0.71; 1.1]
$\beta_{\mathrm{Interrogative}:\mathrm{LongNP}}$	1.51	0.43	[0.65; 2.35]

*Note*. By‐item and by‐participant random intercepts and slopes. Reference level of the predictors is set to “Short” and “Declarative.” Negative estimates for predictors correspond to an acceptability decrease.

We found that baseline conditions were rated higher overall, with the declarative construction being rated higher ($R_{\mathrm{Declarative}-\mathrm{Baseline}}$ = 6.64) than the interrogative construction ($R_{\mathrm{Interrogative}-\mathrm{Baseline}}$ = 6.35). The short‐condition sentences were rated lower than these baselines, again with the declarative construction rated higher ($R_{\mathrm{Declarative}-\mathrm{ShortNP}}$ = 5.52) than its interrogative counterpart ($R_{\mathrm{Interrogative}-\mathrm{ShortNP}}$ = 4.54). Finally, long‐condition sentences were rated lower overall, this time with no major difference between declarative ($R_{\mathrm{Declarative}-\mathrm{LongNP}}$ = 4.11) and interrogative versions ($R_{\mathrm{Interrogative}-\mathrm{LongNP}}$ = 4.30).

Our model provided compelling evidence for an interaction effect between NP object length and clause type (E($\beta_{\mathrm{Interrogative}:\mathrm{LongNP}}$) = 1.51; *SD* = 0.43; 95% CrI = [0.65, 2.35]; *p*($\beta_{\mathrm{Interrogative}:\mathrm{LongNP}}>0$) = 1). While declarative clauses with long NP objects were rated lower than declarative clauses with short NP objects, no difference was found between the two interrogative clauses with short and long NP objects.

### 3.6 Discussion

Experiment 2 allowed us to test the effect of varying filler‐gap distances to disentangle dependency length complexity predictions under no‐ECs and +ECs analyses. To do so, we compared ditransitive constructions in *wh‐*interrogative sentences, using materials motivated by Pickering & Barry (1991) for contrasts like those in (28).

Our results did not support the prediction of the +ECs theories. Indeed, +ECs theories incorrectly predicted that an increase of the length of the NP object would trigger an acceptability reduction in interrogative structures, similar to what we found for declarative structures. In contrast, we found that varying the NP object length had no effect on acceptability for the interrogative structures. Thus, our results support theories with no‐ECs: such theories correctly predict that keeping filler‐head distances constant do not affect acceptability. Overall, these results suggest that the relevant distance to predict acceptability is the filler‐head distance, not the filler‐gap distance, and indeed, that there is no need for gaps at all.

In an oral presentation of this work, it was suggested to us that perhaps the absence of filler‐gap distance effect in (50b), compared to (50a) below, is due to the possibility that the empty category is actually located between the NP *the students* and the relative clause. If this were the case, then the filler‐gap distance would be constant across the two conditions, in the same way as the filler‐head distance. However, this would imply that the relative clause modifying the recipient NP is actually extraposed. This seems unlikely given its restrictive reading, which generally precludes extraposition (Kiss, 2005; Manninen, 2002; Pollard & Sag, 1994), and the fact that the declarative version of the extraposition (50c) is less acceptable than the nonextraposed version. We, therefore, think that this analysis is unlikely.

(50)
  a. [Which prize] did Mary give the students *e* after they answered the question correctly without getting any help?
  b. [Which prize] did Mary give the students **??** *e* who could answer the question correctly without getting any help *e*?
  c. ↓ Mary gave the students [a prize] who could answer the question correctly without getting any help.

## 4 General discussion

**Fig. 4:**
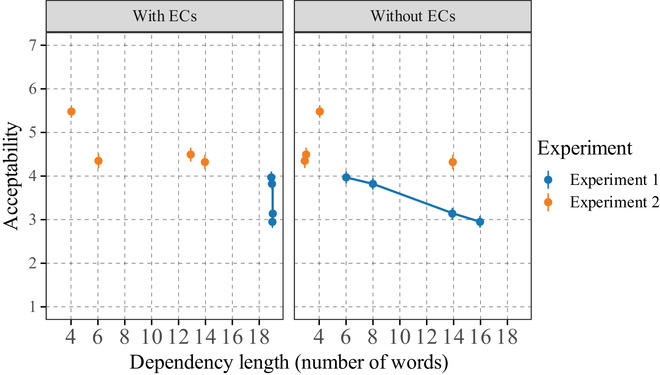
Relation between dependency length and acceptability across two experiments. We include the short and long interrogative versions from E2, which are roughly matched for length with the items from E1. We omit the declarative conditions, as they do not distinguish the two theories, and the baseline conditions, as they are much shorter sentences (acceptability ratings are highly sensitive to sentence length: longer sentences being rated less acceptable, as long as they are grammatical).The theory without empty categories—right panel—shows a close relationship between dependency length and acceptability rating: as dependency lengths get longer, the ratings get lower. In contrast, the theory with empty categories—left panel—shows no correlation with dependency length: the acceptability varies, but dependency length does not predict these ratings.

In two acceptability judgment experiments, we disentangled the effect of filler‐head and filler‐gap distances in the acceptability of long‐distance dependencies. The results are summarized in Fig. 4. This figure presents the relation between dependency length and acceptability depending on the analysis: with or without empty categories.

In Experiment 1, we used nested relative clauses varying filler‐head distance and keeping filler‐gap distance constant. We showed that longer filler‐head distances induce an acceptability decrease. While the no‐ECs analysis predicts this effect, the +ECs one incorrectly predicts no such effects. As shown in the blue dots in the left panel in Fig. 4, the +ECs analysis assumes a fixed dependency length, which does not account for the acceptability differences. In Experiment 2, we used ditransitive verbs in *wh*‐interrogative clauses to vary filler‐gap distance with a constant filler‐head distance. We found that longer filler‐gap distances do not affect acceptability. This is again predicted by the no‐ECs theories, because filler‐head distances are constant. In contrast, +ECs theories incorrectly predict an effect of the filler‐gap distance increase. This absence of effect is shown by the orange dots on the left panel of Fig. 4, where the dependency length increase assumed in +ECs structures does not affect acceptability. In contrast, assuming structures without ECs nicely predicts a negative correlation between dependency length and acceptability: the longer the dependency, the lower the structure's acceptability. This can be seen in the right panel of Fig. 4, where the correlation takes the form of a failing slope. This result is consistent with previous literature on the correlation between dependency length and acceptability in English (Gibson & Thomas, 1997; Gibson, 1998).

Taken together, the results from Experiments 1 and 2 thus show that the filler‐head distance, and not the filler‐gap distance, is relevant to predict acceptability. Dependency length effects are thus more compatible with structures without ECs mediating filler‐gap dependencies. These data, therefore, provide evidence against theories with such ECs, as suggested by Pickering & Barry (1991). One obvious conclusion then is that there might not be such ECs in syntactic representations.

One alternative proposed by Gibson & Hickok (1993) is that perhaps empty categories associated with fronted fillers can be posited as soon as enough information is provided to do so, so that there is not a DLT cost associated with them once their licensor is encountered. Gibson & Hickok (1993) suggested a processing principle called First Resort Gap‐Positing:

(51) **First Resort Gap‐Positing**: Given a filler $\gamma_{i}$ in the structure for the current input string, attach an empty category $\alpha_{i}$ in a position P iff
  a. P is fully licensed by applicable modules of the grammar (e.g., $\bar{X}$‐theory, θ‐theory, Case theory, etc.) and
  b. P and the $\gamma_{i}-\alpha_{i}$ complex are compatible with respect to syntactic category.

This principle allows empty categories to be posited as soon as they are licensed. This means when comprehenders encounter a sentence like (32a) —an easy‐to‐process item from Experiment 1 (repeated below)—they may have a representation as in (52) when encountering the verb *put*. At this point, the empty category related to *on which* has already been posited.

(32a) This is the saucer [on which] Mary put the cup [into which] I poured the tea.

(52) This is the saucer [on which]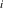 Mary [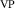 put [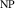 …] [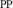 $e_{i}$]]

Further, incoming lexical material can then be added before the empty category. Nothing prevents such a procedure, although it necessitates more complicated structure building operations.

While this kind of a stipulation is always possible to save a theory, we would normally want independent evidence for such a claim. None is provided.^8^ Moreover, this proposal still requires a distance‐based cost theory similar to the DLT to explain acceptability patterns with empty categories (Gibson & Hickok, 1993, p. 158–159), but it has no independent evidence. It amounts to accept the prevalence of the filler‐head distance in explaining acceptability patterns in long‐distance dependencies.

Ultimately, adopting a no‐ECs structure thus allows one to make straightforward predictions under a dependency length complexity approach, without additional stipulations. By contrast, adopting a +ECs structure implies accepting two theoretical complexities: more complex syntactic structures (as they include empty categories) and more complex parsing principles (as they require an EC‐specific principle on top of a DLT‐like principle). This is why adopting no‐ECs structures seems more parsimonious (Pickering, 1993), even if +ECs accounts can be maintained in principle (Gorrell, 1993).

Phillips & Wagers (2012) have suggested that comparing the complexities of grammars with and without transformations is difficult, because there are invariably many details of each grammar that are not discussed, which could contribute to the overall complexity. In particular, they think that grammars without transformations have other complexity somehow built in to them. But, in contrast to Phillips & Wagers (2012)'s claim, it is the transformational theories that are underspecified. Nontransformational theories have been constructed with broad‐coverage grammars, and hence are fully specified. For example, Head‐driven Phrase Structure Grammar (HPSG, Sag, Wasow, & Bender, 1999) received implementations covering a wide range of English (Flickinger, 2000, 1999), German (Crysmann, 2003; Müller & Kasper, 2000), or Japanese (Siegel & Bender, 2002; Siegel, 2000, 2007) constructions, among other languages, and this has led to the development of syntactically annotated corpora—treebanks, such as the LinGO Redwoord corpus (Oepen, Flickinger, Toutanova, & Manning, 2004). There also exist broad‐coverage grammar implementations of other frameworks, such as Combinatory Categorial Grammar (CCG, Steedman, 2001; Hockenmaier & Steedman, 2002; Hockenmaier, 2003, a. o.), Dependency Gramamr (De Marneffe, Manning, Nivre, & Zeman, 2021), and Lexical Functional Grammar (LFG, Bresnan, 1982a; Crouch et al., 2011). Dependency grammar has been used to cover over 100 languages through the Universal Dependency project (De Marneffe et al., 2021; Nivre et al., 2016, 2020). In contrast, there are no broad‐coverage implementations of transformational theories; such implementations are restricted to a few constructions within a single language (e.g., Stabler, 1997; Graf, 2021). Most attempts to implement transformational theories are actually phrase‐structure grammars with no transformations at all, or with operations that provide a superficial similarity to transformations but which are actually quite different from a theoretical perspective (Müller, 2023, p. 120–121). For example, in Stabler (1987)'s Government‐Binding‐inspired (Chomsky, 1981) grammar for English, transformation‐like operations apply before the general phrase‐structure rules to parse sentences. One such operation, auxiliary inversion, specifically targets auxiliaries appearing in first position to find their base position after the subject. This is radically different from movement, as it is assumed in Chomsky's Government‐Binding theory, since this process depends on linear order, not structure. Furthermore, the process should be construction‐general, and not restricted to the inversion of an auxiliary. So, contrary to Phillips & Wagers (2012)'s claim, it is transformational grammars that are underspecified. They may be even more complex than suggested here. For example, we showed that, under a transformational grammar, the number of possible dependency structures S associated with a string of n words is at least $S\geq n^{n-1}\times n^{n}$, which is much larger than under a non‐transformational grammar ($S=n^{n-1}$). Not only that but the transformational estimate omits, for instance, that words can move several times, and that whole phrases can move as well. So, transformational grammars are indeed more complex than nontransformational grammars: a theory without transformations has strictly less to learn.

What then is the evidence for empty categories associated with movement in linguistic theories? As discussed in the introduction, the original motivation for such empty elements was provided by Chomsky (1957, 1965) in terms of the simplicity of the grammar: Chomsky thought that the simplest way to explain auxiliary inversion in English interrogatives was in terms of movement from the “base” position in a declarative. But, we now know that there are alternative analyses for what might superficially seem to be “movement” phenomena. For instance, HPSG accounts for the English auxiliary inversion using appropriate feature specifications of lexical entries and phrase‐structure rules (Sag et al., 2020). Moreover, Chomsky (1971) later noted that a transformational theory comes with learning problems: it is hard for a child to converge on the correct grammar, given the ambiguity of representations that is created by having the possibility of moving words and phrases around. We elaborated this issue in the introduction, demonstrating that the learning problem associated with transformations is astronomically worse than that associated with learning just the dependency structures. And finally, the nontransformational theories are more compatible with what structures are possible, with respect to auxiliary verbs in English, as summarized in the introduction. The same applies to other structures that have been examined in detail, such as the passsive. The current paper shows that filler‐gap dependencies are also better explained by a theory without transformations.

To conclude, the empirical results of our experiments are most easily compatible with analyses of filler‐gap constructions without empty categories mediating long‐distance dependencies. Even if empty categories could still be maintained in principle, at the cost of added stipulations, we argue that syntactic complexity phenomena are better explained without them. This conclusion is consistent with previous studies (Pickering & Barry, 1991; Pickering, 1993).

## Conflicts of interest

The authors do not have any conflicts of interest to disclose.

## Figures and Tables

**Fig. A.1:**
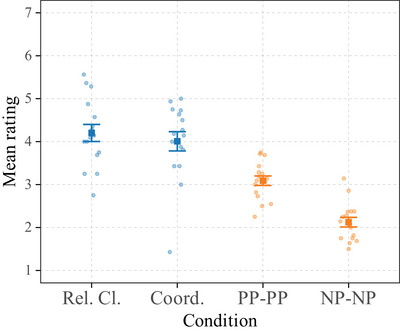
Mean ratings by item (dots) and by condition (squares). Participants = 38, items = 16, observations = 608. Error bars represent standard error.

## Notes

1 Following standard notation in the literature, we indicate the source position with an underscore, and we coindex this position and the position to which it moves with an index, here *i*.

2 Phillips and Wagers (2012) have suggested that comparing the complexities of grammars with and without transformations is difficult, because there are invariably many details of each grammar that are not discussed, which could contribute to the overall complexity. In particular, they think that grammars without transformations have other complexity somehow built in to them. But, in contrast to Phillips & Wagers (2012)'s claim, it is the transformational theories that are underspecified. Nontransformational theories have been constructed with broad‐coverage grammars, and hence are fully specified. Some examples are the English Resource Grammar (Flickinger, 1999) or the Jacy grammar for Japanese (Siegel, 2007), belonging the larger delph‐in project framed in HPSG. Other examples include Universal Dependency parsers, covering more than 100 langugages (De Marneffe & Nivre, 2019; Qi, Zhang, Zhang, Bolton, & Manning, 2020) or XLE grammars framed in LFG (Crouch et al., 2011). So, contrary to Phillips & Wagers (2012), we think that transformational grammars are indeed more complex than nontransformational grammars, exactly in the sense discussed here: a theory without transformations has strictly less to learn.

3 This usage of *ought* is apparently acceptable to older British English speakers, suggesting a usage shift over time.

4 We adopt the term *gap* in a theoretically neutral way, such that the canonical position of the complement is not lexicalized: the transitive verb *read* in (23) appears without a lexical element in the canonical complement position and a complement cannot be expressed lexically in that position (i).

(i) *Which book did Mary say you have read the newspaper tomorrow?

However, the question whether there is a syntactic object in this canonical position remains. This is what we address in this paper.

5 We take the down‐arrow notation from Miller (2013) to indicate an acceptability reduction in a pair of sentences.

6 These implausible combinations could themselves trigger a difficulty, since the default assumption is that NP fillers followed by a subject are objects (Omaki et al., 2015). However, this possibility would lead us to our first point: there should be no difference between the first and second fillers.

7 We thank a reviewer for this suggestion.

8 A reviewer suggests that studies found in Aoshima et al. (2004) and Omaki et al. (2015) are evidence for the principle in (51). These papers do provide evidence for predictive parsing strategies in long‐distance dependency processing, alongside head‐ and filler‐driven strategies. However, they do not address the question whether those predictions involve incomplete dependencies or empty elements. In general, effects of pre‐head processing can be taken as evidence for a general predictive dependency building rather than empty‐category positing.

## Appendix A Experiment A

Experiment A was a first experiment we designed to evaluate the relative complexity of PP‐PP and NP‐NP sentences (from Pickering & Barry (1991) and tested in Experiment 1). We compared them to two baseline conditions expressing the same content with less complex structures: a simple relative clause and a coordination. However, in this experiment, we did not control the effect of each filler‐head distance separately, because we compared PP‐PP and NP‐NP conditions directly, without the intermediate PP‐NP and NP‐PP conditions we used in Experiment 1.

### A.1 Materials

We created 16 critical items in each of four conditions, as in (38). The sentences in PP‐PP and NP‐NP conditions are a subset of the sentences used in Experiment 1.

(53) a. PP‐PP: This is the saucer on which Mary put the cup into which she poured the milk.
  b. NP‐NP: This is the saucer which Mary put the cup which she poured the milk into on.
  c. Coordination: Mary put the cup on the saucer and she poured the milk into the cup.
  d. Relative clause: Mary poured the milk into the cup which she put on the saucer.

All items were constructed following the structure in (53), with the same controls as in Experiment 1: as many female names as male proper names, all common nouns refer to inanimate entities, all verbs use PP‐locative complements. All items can be found on the OSF project of this study (https://osf.io/dyfqh/). The experiment also included two practice items at the beginning of the task and 48 fillers containing other types of structure (temporal adjunct clauses, interrogative and exclamative clauses, passive voice sentences). The experiment thus consisted of 66 trials in total.

### A.2 Procedure

We ran an acceptability judgment task on the Ibex Farm platform, recruiting 40 native US English speakers from Prolific. Participation was anonymous, voluntary, and paid ($5). All participants gave their consent for the data analysis, and they can retract their data using their Prolific ID.

The experiment was set online. Participants had to read sentences on their screen and give a rating on a 1–7 Likert scale (“unacceptable” to “acceptable”). Each sentence was displayed one by one on the screen. Half of the sentences were followed by a yes/no comprehension question. At the beginning of the experiment, participants had to fill out a sociolinguistic questionnaire (native language(s), country of L1 acquisition, age, gender, other known languages).

Presentation of critical items and filler was randomized. We used the Latin Square method for the presentation of critical items, each participant seeing each item in only one condition.

### A.3 Participants

Based on their accuracy to comprehension questions, we excluded two participants who got 71% correct responses comprehension questions, less than the 80% threshold. We thus analyze data for 38 participants: 19 women, 15 men, and 4 nonbinary/agender people. Mean age is 35 years (*SD* = 14.3, Max = 70 years, Min = 18 years). Participants' average accuracy score for comprehension questions is 96% (*SD* = 0.02).

### A.4 Statistical analysis

We analyze acceptability judgment data running a Bayesian cumulative ordinal regression model, with the *logit* link function on R (package *brms*, Bürkner (2017)).

We fitted a model to sentence rating with sentence structure as predictors (coordination, relative clause, clefted PPs, or clefted NPs), taking “relative clause” as the reference level. We treatment‐coded the categorical predictors (coding 0 for the reference level, 1 for the other levels). We included by‐participant and by‐item intercepts and slopes as random effects. We used weak informative priors for fixed effects (Normal distribution with μ=1;σ=0) and used the default priors for the thresholds and the standard deviation of random effects (Student's *t*‐distribution with μ=0;σ=2.5;ν=3).

We are interested in the following three differences:

(54) We expect the PP‐PP condition to be rated lower than the relative clause baseline: $\beta_{\mathrm{PP}-\mathrm{PP}}<0$
(55) We expect the PP‐PP condition to be rated lower than the coordination baseline: $\beta_{\mathrm{Coordination}}>\beta_{\mathrm{PP}-\mathrm{PP}}$
(56) We expect the NP‐NP condition to be rated lower than the PP‐PP condition: $\beta_{\mathrm{NP}-\mathrm{NP}}<\beta_{\mathrm{PP}-\mathrm{PP}}$

### A.5 Results

Descriptive results are shown in Fig. A.1, and the summary of the model's posterior distributions are given in Table A.1.

**Table A.1:** Summary of the population‐level posterior distributions of the ordinal model

Parameter	Estimate	Standard deviation	95% Credible interval
**Thresholds**			
$\tau_{1}$	−4.63	0.44	[−5.5; −3.78]
$\tau_{2}$	−2.39	0.40	[−3.17; −1.6]
$\tau_{3}$	−1.05	0.39	[−1.79;−0.27]
$\tau_{4}$	0.19	0.38	[−0.56; 0.95]
$\tau_{5}$	1.63	0.40	[0.87; 2.43]
$\tau_{6}$	3.36	0.43	[2.54; 4.24]
**Predictors**			
$\beta_{\mathrm{Coordination}}$	−0.16	0.40	[−0.95; 0.63]
$\beta_{\mathrm{NP}-\mathrm{NP}}$	−3.22	0.48	[−4.11; −2.27]
$\beta_{\mathrm{PP}-\mathrm{PP}}$	−1.66	0.32	[−2.3;−1.01]

By‐item and by‐participant random intercepts and slopes. Reference level of the predictors is set to the “Relative clause” condition. Negative estimates for predictors correspond to an acceptability decrease.

We found that the two baseline conditions with relative clauses and coordinations were rated quite similarly: E($R_{\mathrm{RelativeClause}}$) = 4.3, 95% CrI = [3.76; 4.79] and E($R_{\mathrm{Coordination}}$) = 4.19, 95% CrI = [3.49; 4.86]. The PP‐PP condition is rated lower than the baselines (E($R_{\mathrm{PP}}-\mathrm{PP}$) = 3.17, 95% CrI = [2.63; 3.73]), and our model gives compelling evidence for these differences: the PP‐PP condition is rated lower than the relative clause condition (E($\beta_{\mathrm{PP}-\mathrm{PP}}$) = −1.66; *SD* = 0.32; 95% CrI = [−2.3,−1.01]; *p*($\beta_{\mathrm{PP}-\mathrm{PP}}<0$) = 1) than the coordination condition (E($\beta_{\mathrm{Coordination}}-\beta_{\mathrm{PP}-\mathrm{PP}}$) = 1.5; *SD* = 0.48; 95% CrI = [0.56, 2.43]; *p*($\beta_{\mathrm{Coordination}}>\beta_{\mathrm{PP}-\mathrm{PP}}$) = 1). Finally, the NP‐NP condition is rated the lowest (E($R_{\mathrm{NP}}-\mathrm{NP}$) = 2.26, 95% CrI = [1.77; 2.89]), and we found compelling evidence that it is rated lower than the PP‐PP condition (E($\beta_{\mathrm{NP}-\mathrm{NP}}-\beta_{\mathrm{PP}-\mathrm{PP}}$) = −1.57; *SD* = 0.53; 95% CrI = [−2.6,−0.47]; *p*($\beta_{\mathrm{NP}-\mathrm{NP}}<\beta_{\mathrm{PP}-\mathrm{PP}}$) = 1).

Interestingly, the effect size of the NP‐NP versus PP‐PP difference is equivalent across the two experiments: E($\eta_{\mathrm{NP}-\mathrm{NP}}-\eta_{\mathrm{PP}-\mathrm{PP}}$) = −1.57; 95% CrI = [−2.6,−0.47] for Experiment A and $E(\eta_{\mathrm{NP}}-\mathrm{NP}-\eta_{\mathrm{PP}}-\mathrm{PP})=$−1.67; *SD* = 0.31; 95% CrI = [−2.28,−1.05] for Experiment 1.
